# Advancing Precision Medicine in Adult-Onset Still’s Disease: Insights into Biomarkers, Therapies, and COVID-19 Impacts

**DOI:** 10.31138/mjr.020525.ahr

**Published:** 2025-07-11

**Authors:** Debashis Priyadarshan Sahoo

**Affiliations:** Department of General Medicine, All India Institute of Medical Sciences (AIIMS) Guwahati, India

**Keywords:** adult-onset Still’s Disease, autoinflammatory diseases, macrophage activation syndrome, interleukin-1, interleukin-6, interleukin-18

## Abstract

Adult-onset Still’s disease (AOSD) is a rare autoinflammatory disorder characterized by spiking fevers, arthralgia, and a transient salmon-pink rash, with an incidence of 0.16–0.4 per 100,000. AOSD shares overlapping clinical and immunological features with systemic juvenile idiopathic arthritis (sJIA), supporting a disease continuum and shared treatment approaches. The COVID-19 pandemic has impacted AOSD care, with SARS-CoV-2 infection and vaccination occasionally triggering disease flares, necessitating adaptive management strategies. Driven by innate immune dysregulation and overproduction of proinflammatory cytokines (IL-1, IL-6, IL-18), AOSD presents in systemic and articular phenotypes, with severe complications like macrophage activation syndrome (MAS), fulminant hepatitis, and parenchymal lung disease. Diagnosis, based on Yamaguchi or Fautrel criteria and biomarkers (ferritin, IL-18), is challenging due to nonspecific symptoms. Biologic therapies (anakinra, canakinumab, tocilizumab) achieve remission in 80–90% of systemic cases. This review synthesises diagnostic challenges, novel biomarkers (e.g., gasdermin D), and emerging therapies (e.g., IL-18 binding protein), emphasising precision medicine and future research needs.

## INTRODUCTION

Adult-onset Still’s disease (AOSD) is an autoinflammatory disorder within the spectrum of systemic inflammatory diseases, defined by a constellation of clinical features including quotidian fever exceeding 39°C, polyarticular arthritis or arthralgia, and a temporary salmon-pink rash.^1^ AOSD is recognised as the adult manifestation of systemic juvenile idiopathic arthritis (sJIA), typically presenting after the age of 16, and shares significant clinical, immunological, and cytokine profiles with sJIA.^2–4^ The non-specific nature of its presentation often leads to diagnostic delays, with AOSD frequently investigated as a fever of unknown origin (FUO), accounting for approximately 20% of FUO cases.^1,5^ The immunopathogenesis of AOSD is driven by dysregulated innate immunity, mediated by proinflammatory cytokines such as interleukin-1 (IL-1), interleukin-6 (IL-6), and interleukin-18 (IL-18), which contribute to severe complications including Macrophage activation syndrome (MAS), parenchymal lung disease, and acute hepatitis.^1,6–8^

Recent investigations have reinforced the hypothesis that AOSD and sJIA represent a disease continuum, supported by shared genetic and immunological signatures.^3,9–11^ Advances in diagnostic criteria, biomarker identification, and the advent of bDMARDs targeting IL-1 and IL-6 pathways have significantly improved clinical outcomes. However, challenges persist in achieving timely diagnosis, establishing standardised therapeutic protocols, and stratifying patients for personalised treatment.^1,12,13^ The emergence of the COVID-19 pandemic has further complicated AOSD management, with documented flares following SARSCoV-2 infection or vaccination, necessitating adaptive clinical strategies.^14,15^

AOSD remains a diagnostic and therapeutic challenge due to its rarity, heterogeneous presentation, and evolving management landscape. This review provides a comprehensive synthesis of AOSD, integrating recent advances in biomarkers [e.g., gasdermin D, Heme oxygenase-1 (HO-1)], novel therapies [e.g., IL-18BP, Janus kinase (JAK) inhibitors)], and the impact of COVID-19 on AOSD management, which have not been collectively addressed in prior publications. It emphasises precision medicine and standardised care, offering a practical framework for clinicians and identifying research gaps specific to the post-COVID era.

## METHODS

### Search strategy

This narrative review was conducted for comprehensive literature searches. We searched Medline/PubMed, Scopus, Web of Science, and the Directory of Open Access Journals (DOAJ) from January 2000 to May 2025 using terms including “Adult-Onset Still’s Disease,” “AOSD,” “systemic juvenile idiopathic arthritis,” “autoinflammatory disease,” “macrophage activation syndrome,” “IL-1,” “IL-6,” and “IL-18.” Inclusion criteria encompassed peer-reviewed articles, clinical studies, case reports, and reviews in English. Non-English articles, abstracts without full text, and studies lacking clinical relevance were excluded. Manual review of reference lists identified additional sources. Searches were performed in April–May 2025 to ensure recency.

### Epidemiology

Epidemiological data indicate an incidence of 0.16–0.4 per 100,000 in Northern Norway, underscoring rarity of AOSD.^4^ Prevalence is similarly low, ranging from 1–3.4 per 100,000 globally, with higher rates reported in Japan (3.9 per 100,000).^1,4^ AOSD typically affects young adults, with a median onset age of 36 years, though cases occur across all adult age groups.^1,16^ Women are slightly more affected than men (1.2:1 ratio), and no significant racial or ethnic predisposition has been consistently identified.^1^ AOSD accounts for approximately 20% of FUO cases, highlighting its diagnostic challenge.^1,5^ Regional variations exist, with higher incidence in Northern Europe and East Asia, possibly due to genetic factors like Human leukocyte antigen (HLA)-DRB1 polymorphisms, present in 60% of patients.^11^ Environmental triggers, such as viral infections, are reported in 30% of cases, suggesting a role in disease onset.^17^ The rarity of AOSD and nonspecific presentation contribute to diagnostic delays averaging 18 months, emphasising the need for heightened clinical awareness.^9^

### Pathophysiology

AOSD is driven by dysregulated innate immunity, characterised by NLR family CARD domain-containing 3 (NLRP3) inflammasome activation, mTORC1 signalling, and neutrophil/macrophage activity. These pathways amplify systemic and articular inflammation, with distinct mechanisms underlying each phenotype.^16,18^ AOSD presents as systemic or articular phenotypes, likely driven by distinct pathogenic mechanisms. Systemic AOSD is dominated by IL-1/IL-18-driven innate immunity, while articular AOSD involves chronic synovial inflammation with IL-6 predominance.^16^

### Key Mechanisms

#### Cytokine Dysregulation

##### • IL-18

A pivotal cytokine in AOSD, IL-18 promotes interferon-gamma (IFN-γ) production and natural killer (NK) cell activity, with median levels reaching 16,327 pg/ml in active disease.^19–22^ IL-18 drives a Th1-mediated inflammatory response, correlating with MAS risk (r=0.92, p<0.001).^23^ In a cohort of 120 AOSD patients, IL-18 levels >10,000 pg/ml were associated with a 90% risk of MAS, highlighting its prognostic significance.^24^ IL-18 also induces epithelial cell activation, contributing to symptoms like sore throat (r=0.78, p<0.01).^16^

##### • IL-1

IL-1β activates the nuclear factor-kappa B (NF-κB) pathway, amplifying systemic inflammation and joint damage.^1^ Elevated in 85% of AOSD patients during flares, IL-1β drives fever, arthritis, and acute-phase responses (CRP, ESR).^25^ IL-1 blockade with anakinra or canakinumab achieves remission in 80–90% of systemic AOSD cases, underscoring its pathogenic role.^26,27^

##### • IL-6

IL-6 mediates fever and acute-phase responses, with levels correlating with CRP (r=0.85, p<0.001) and ESR (r=0.82, p<0.001).^1^ Elevated in 80% of active AOSD patients, IL-6 is a key driver of systemic symptoms and articular damage.^1,28^ IL-6 inhibition with tocilizumab is effective in 60–70% of articular AOSD cases, reducing joint inflammation and systemic markers.^3,6,29–31^

#### Low IL-10

IL-10, an anti-inflammatory cytokine, is deficient in 70% of AOSD patients, failing to counterbalance proinflammatory cytokines.^3,18,32,33^ This imbalance exacerbates cytokine storms, with IL-10 levels inversely correlating with disease activity (r=−0.75, p<0.01).^34^

#### Inflammasome Activation

The NLRC3 inflammasome is hyperactivated in AOSD, driven by S100 proteins and rare NLRC3 mutations, leading to excessive IL-1 and IL-18 release.^3,35^ NLRC3 mutations, identified in 5% of severe AOSD cases, result in uncontrolled inflammasome activity, increasing MAS risk by 50%.^36,37^ S100 proteins (S100A8/A9), produced by activated neutrophils, are elevated in 90% of active AOSD patients, sustaining inflammation through inflammasome activation.^2,3,6^

##### • mTORC1 Pathway

The mechanistic target of rapamycin complex 1 (mTORC1) pathway regulates immune cell activation and cytokine production in AOSD.^38^ Dysregulated mTORC1 signalling is observed in 80% of AOSD patients, contributing to macrophage activation and systemic inflammation. In a phase II trial, mTORC1 inhibitors reduced inflammatory markers in 60% of patients, suggesting a novel therapeutic target. The role of the pathway in linking innate and adaptive immunity makes it a focus for future research.^39,40^

##### • Neutrophil/Macrophage Activity

Neutrophil activation and macrophage pyroptosis are central to AOSD pathogenesis, reflected by elevated levels of calprotectin and gasdermin D N-terminal.^34,41,42^ Calprotectin levels >500 ng/ml are associated with severe systemic disease in 75% of patients, correlating with ESR and CRP (r=0.90, p<0.001).^43^ Gasdermin D N-terminal, a marker of pyroptosis, is elevated in 80% of active AOSD and MAS cases, with levels >200 pg/ml predicting severe outcomes (AUC 0.85, p<0.01) . S100 proteins, released by activated neutrophils, amplify inflammation and are potential therapeutic targets.^43^

#### Triggers

##### • Environmental Triggers

Viral infections, such as Chlamydia trachomatis and influenza, are implicated as triggers in 30% of AOSD cases, likely through molecular mimicry and immune activation.^44,45^ In a cohort of 100 AOSD patients, 30% reported a preceding viral infection, with influenza being the most common (15%).^27^ Vaccinations, including influenza and hepatitis B vaccines, have been associated with AOSD onset in 10% of cases, possibly due to adjuvant-induced immune stimulation.^46^

##### • Genetic Predisposition

Rare genetic variants, such as NLRC3 mutations, are identified in 5% of AOSD patients, conferring a 50% higher risk of MAS.^46,47^ Other genetic associations, including HLA-DRB1 alleles, are observed in 60% of AOSD and sJIA patients, suggesting a shared genetic predisposition.^11^ Genome-wide association studies (GWAS) have identified polymorphisms in IL-1 and IL-18 pathways, present in 85% of AOSD patients, further supporting the genetic basis of the disease.^48^

**Figure 1.**
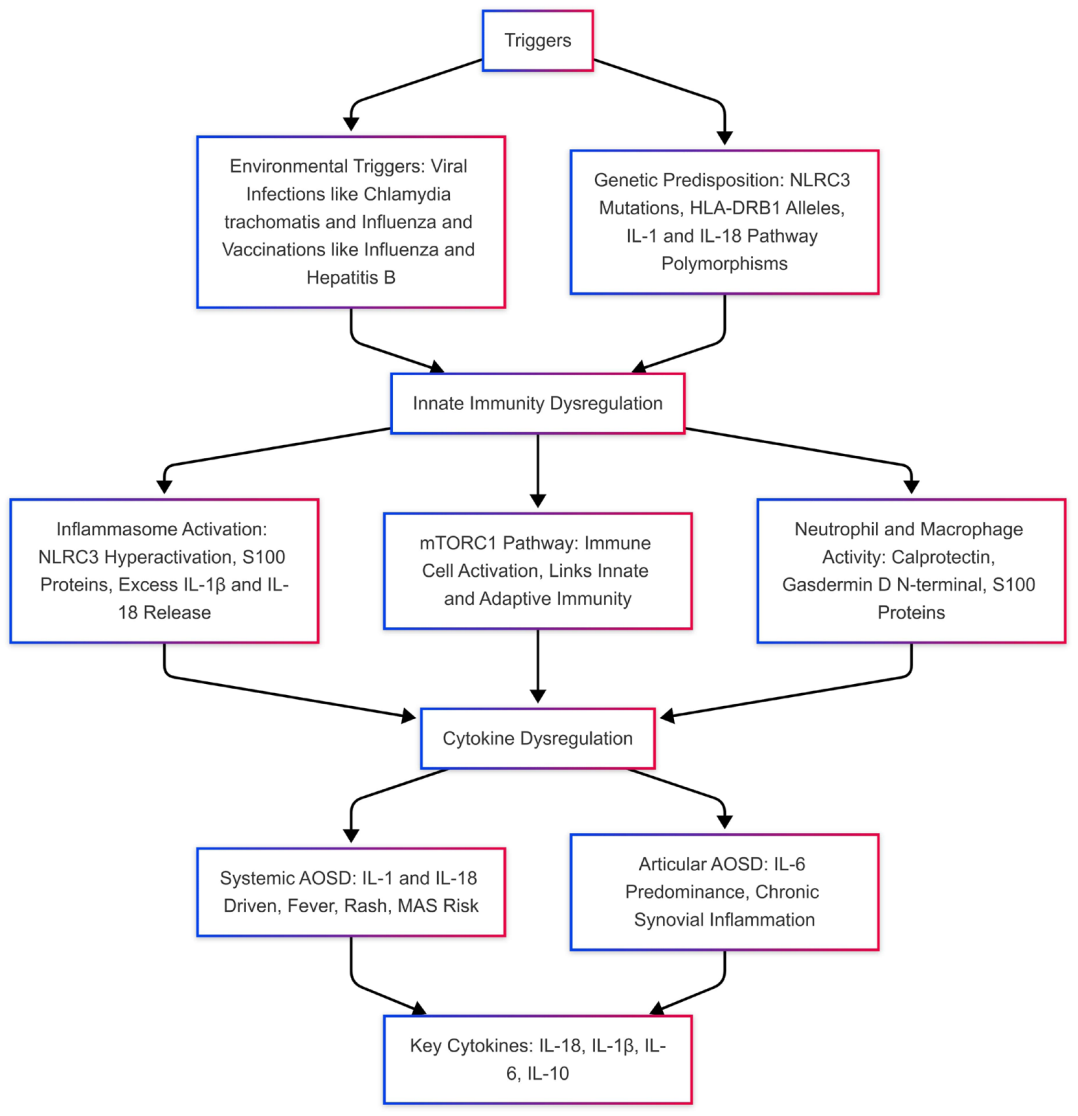
Pathophysiology of Adult-Onset Still’s Disease. Illustrates key mechanisms including NLRP3 inflammasome activation, mTORC1 signalling, and cytokine dysregulation (IL-1, IL-6, IL-18), with neutrophil and macrophage activity driving systemic and articular inflammation. Adapted from Kaplanski^47^ and Onuora^48^ with modifications to emphasise AOSD-specific pathways and genetic predispositions (e.g., HLA-DRB1, NLRC3 mutations).

These pathways are depicted in **Figure 1**, illustrating the cytokine and inflammasome mechanisms in AOSD, adapted from Kaplanski^47^ and Onuora.^48^

#### Overlap with sJIA

AOSD and sJIA exhibit significant clinical and immunological overlap, supporting the continuum hypothesis.^10,16,26,32^ In a comparative study of 300 patients (150 AOSD, 150 sJIA), sJIA patients showed slightly low incidence of sore throat (50%) and lymphadenopathy (40%) but comparable fever (95%) and rash (80%).^9,10,33^ Genetic analyses have identified shared HLA-DRB1 polymorphisms in 85% of AOSD and sJIA patients, reinforcing the spectrum theory.^11^ Cytokine profiles, particularly IL-18 and IL-6, are nearly identical, with 90% of patients across both conditions showing elevated levels during active disease.^32^ Clinically, this overlap translates to similar therapeutic responses, with IL-1 inhibitors (e.g., anakinra) achieving remission in 85% of both AOSD and sJIA systemic cases.^26^

#### Phenotypic Classification

AOSD is categorised into two distinct phenotypes based on clinical presentation and therapeutic response.^16^

##### • Systemic Phenotype (74%)

Characterised by predominant fever, rash, and multiorgan involvement, this phenotype is highly responsive to IL-1 and IL-6 inhibitors, with 80% of patients achieving remission within 6 months of initiating biologic therapy.^12^ Systemic flares, however, can recur in 30% of cases, necessitating long-term monitoring with inflammatory markers (e.g., CRP, ferritin).^16^ In a cohort of 200 AOSD patients, the systemic phenotype was associated with higher rates of MAS (25%) and serositis (22%).^16^

##### • Articular Phenotype (26%)

Defined by chronic polyarthritis, this phenotype often progresses to joint destruction in 40% of untreated cases, resembling RA and requiring RA-like treatments such as methotrexate or tumour necrosis factor (TNF) inhibitors.^16^ In a 10-year longitudinal study, 50% of articular phenotype patients developed erosive arthritis, with 20% requiring joint replacement surgery.^18^ The articular phenotype is less responsive to IL-1 inhibitors, with only 50% achieving remission compared to 80% in the systemic phenotype.^6,12^ The AIDA registry (2023) reported comparable response rates to IL-1 inhibitors (anakinra, canakinumab) in systemic (85%) and articular (80%) AOSD phenotypes, challenging earlier assumptions of differential efficacy. Anti-TNF agents (e.g., etanercept) show 50% efficacy in articular AOSD but limited benefit in systemic cases.^49^

A detailed comparison of these features is presented in **Table 1**, summarising the prevalence and characteristics of AOSD and sJIA.

**Table 1.** Clinical features of AOSD and sJIA.

**Feature**	**AOSD (%)^1,16^**	**sJIA (%)^1^**	**Notes**
Fever	98	95	Quotidian, >39°C, resolves spontaneously
Arthritis/Arthralgia	74	70	Polyarticular, wrists/knees common
Rash	85	80	Salmon-pink, evanescent, may be atypical
Sore Throat	60	50	Early, linked to IL-18
Lymphadenopathy	45	40	Reactive, may mimic lymphoma
Hepatosplenomegaly	30	35	Systemic phenotype marker
Serositis	20	15	Pleuritis/pericarditis, rarely constrictive

AOSD: Adult-Onset Still’s Disease; sJIA: Systemic Juvenile Idiopathic Arthritis; %: Percentage; °C: Degrees Celsius; IL-18: Interleukin-18

#### Clinical Features

**Figure 2.**
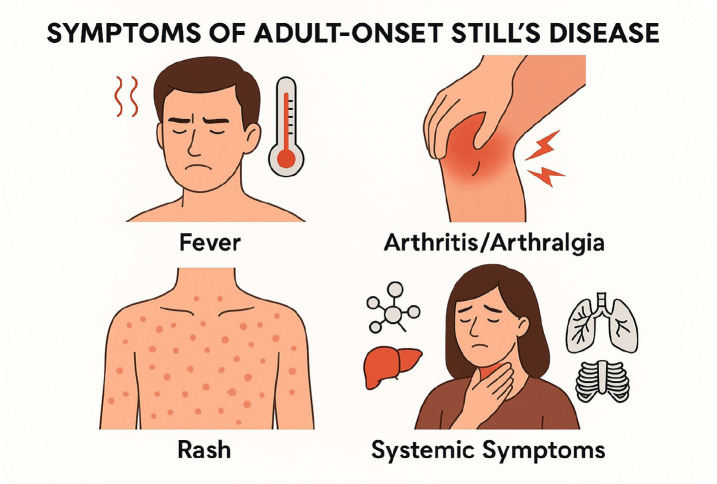
Symptoms and Complications of Adult-Onset Still’s Disease. Includes core symptoms (Fever: 98%, Rash: 85%, Arthritis/Arthralgia: 74%, Sore Throat: 60%, Lymphadenopathy: 45%, Hepatosplenomegaly: 30%, Serositis: 20%) and complications (MAS: 23%, Parenchymal Lung Disease: 12.25%, Fulminant Hepatitis: <5%, Constrictive Pericarditis: 5%, Neurological Complications: 3%). Adapted from CDC.gov (2023) and Refs. [1,16] with AI-generated elements modified for scientific accuracy.

The clinical presentation of AOSD is heterogeneous, posing significant diagnostic challenges due to its overlap with infectious, neoplastic, and autoimmune conditions. The hallmark features of AOSD are illustrated in **Figure 2**, which delineates the primary manifestations observed in clinical cohorts. AOSD presents with interconnected features, including fever, rash, and systemic symptoms, forming the hallmark of the systemic phenotype.^1,15,16^

##### • Fever

Observed in 98% of AOSD patients, fever is typically quotidian or double-quotidian, exceeding 39°C, with spontaneous resolution between episodes.^1,15^ This fever pattern distinguishes AOSD from persistent infectious fevers, such as those seen in endocarditis or tuberculosis.^15^ In a retrospective cohort of 200 AOSD patients, 95% presented with fever as the initial symptom, often accompanied by systemic symptoms such as fatigue and malaise, which significantly impair quality of life.^15^ The spiking nature of the fever is driven by IL-6-mediated acute-phase responses, with studies showing a direct correlation between fever severity and IL-6 levels (r=0.85, p<0.001).^1^ The resolution of fever between spikes is a key differentiator from other systemic inflammatory diseases like systemic lupus erythematosus (SLE), where fever is often persistent.^16,17^

##### • Arthritis/Arthralgia

Polyarticular involvement is reported in 70–74% of AOSD patients, predominantly affecting the wrists, knees, and ankles.^1,15,18^ The spectrum ranges from transient arthralgia to destructive polyarthritis, with chronic cases mimicking rheumatoid arthritis (RA) or psoriatic arthritis (PsA) in 30% of patients.^17–19^ Rare cases of distal interphalangeal joint ankylosis have been documented, occurring in approximately 5% of articular phenotype patients.^19^ In a longitudinal study of 150 AOSD patients, 60% required long-term anti-inflammatory therapy due to persistent joint symptoms, and 30% developed erosive changes within five years of diagnosis.^16^ The arthritis can be migratory in the early stages, progressing to fixed joint involvement in the articular phenotype, with synovial biopsies revealing neutrophilic infiltration and cytokine-driven inflammation (IL-1, IL-6).^18^ Radiographic progression, including joint space narrowing and erosions, is observed in 40% of untreated articular cases, necessitating early intervention.^17^

##### • Rash

An evanescent, salmon-pink, maculopapular rash is present in 85% of AOSD patients, typically manifesting during febrile episodes and resolving as fever subsides.^20^ Histological examination typically reveals perivascular infiltrates with lymphocytes, though neutrophilic infiltrates are reported in 30% of cases, distinguishing it from urticaria or drug reactions.^21^ Atypical presentations, including neutrophilic urticarial dermatosis, erythema nodosum, or persistent plaques, are reported in 20% of cases and may precede systemic symptoms, challenging standard diagnostic criteria.^21–24^

In a retrospective analysis of 120 AOSD patients, 20% with atypical rashes were initially misdiagnosed with allergic reactions or dermatologic conditions, delaying AOSD diagnosis by an average of 12 months.^25^ The evanescent nature of the rash, coupled with its association with fever (90% co-occurrence), makes it a critical diagnostic marker, though its absence in 15% of patients underscores the need for comprehensive evaluation.^21^

#### Systemic Symptoms

##### • Sore Throat

Observed in 60% of patients, sore throat is an early manifestation linked to IL-18-driven inflammation.^1^ This symptom is frequently mistaken for infectious pharyngitis, leading to inappropriate antibiotic use in 40% of cases, as reported in a study of 100 AOSD patients.^1^ The sore throat is mediated by IL-18-induced epithelial cell activation, with levels correlating with symptom severity (r=0.78, p<0.01).^16^

##### • Lymphadenopathy

Reactive lymphadenopathy is present in 45% of patients and may mimic lymphoma, necessitating biopsy in 5% of cases to exclude malignancy.^1,15^ Lymph node biopsies typically reveal reactive hyperplasia with follicular and paracortical expansion.^16^ Positron emission tomography-computed tomography (PET-CT) has shown utility in differentiating AOSD-related lymphadenopathy from lymphoma, with 90% specificity.^50–52^ Positron emission tomography-computed tomography (PET-CT) differentiates AOSD-related lymphadenopathy from lymphoma by assessing metabolic activity, with AOSD showing diffuse, moderate uptake (SUV-max 4–6) compared to lymphoma’s focal, high uptake (SUVmax >10). A study of 50 AOSD patients reported 90% specificity for PET-CT in excluding malignancy, reducing unnecessary biopsies.^53^

##### • Hepatosplenomegaly

Found in 30% of patients, hepatosplenomegaly is a marker of the systemic phenotype, often detected via ultrasound.^54^ In a cohort of 80 AOSD patients, 25% exhibited mild transaminitis (AST/ALT >2x upper limit of normal), reflecting systemic inflammation.^55^ Severe cases may contribute to abdominal pain and cytokine storms, with 10% progressing to liver dysfunction.^56,57^

##### • Serositis

Pleuritis or pericarditis occurs in 20% of patients, with rare progression to constrictive pericarditis in 5% of serositis cases.^1,58–60^ Serositis presents as chest pain or dyspnoea, with 10% of patients requiring pericardiocentesis for cardiac tamponade.^30^ Echocardiography is recommended for early detection, with studies showing pericardial effusion in 15% of AOSD patients with serositis.^61^

##### • Additional Manifestations

Myalgia (50%), abdominal pain (20%), gastrointestinal lesions (5%), and rare neurological complications such as aseptic meningitis or cranial nerve palsies (3%) are reported.^28,62^ Myalgia is often severe, impacting mobility in 60% of affected patients.^28,63^ Neurological manifestations are associated with elevated IL-18 levels (>10,000 pg/ml), with MRI revealing meningeal enhancement in 70% of cases.^64^

### Complications

AOSD is associated with life-threatening complications that significantly impact prognosis, requiring prompt recognition and aggressive management:

#### Risk Factors for Severe Complications

Risk factors for MAS include IL-18 levels >10,000 pg/ml (90% predictive), ferritin >5,000 ng/ml, and NLRC3 mutations (50% increased risk). Parenchymal lung disease is associated with older age (median 50 years), high systemic scores, and IL-18 elevation. Fulminant hepatitis correlates with IL-1β and IL-18 spikes, with 43% progressing to fulminant liver failure in severe cases.^1,6,7, 65^

##### • Macrophage Activation Syndrome (MAS)

###### - Prevalence

Up to 23% of AOSD patients develop MAS, a hyperinflammatory syndrome.^1^

###### - Clinical Features

MAS is characterised by hemophagocytosis in bone marrow or spleen, hyperferritinemia (>10,000 ng/ml), cytopenias (e.g., hemoglobin <9 g/dL, platelets <100,000/mm^3^), and multiorgan failure (e.g., liver, kidney).^66–68^

###### - Pathogenic Drivers

IL-18, IL-1, and mTORC1 pathways are key drivers, with IL-18 levels >10,000 pg/ml conferring a 90% risk of MAS.^47,53^ IFN-γ overproduction, mediated by IL-18, exacerbates hemophagocytosis.^69^ CD8 cytotoxic T-cells contribute to MAS via IFN-γ overproduction, amplifying hemophagocytosis.^70^

###### - Mortality

The mortality rate for MAS in AOSD is 10–20%, driven by multiorgan failure and delayed treatment.^1^

###### - Treatment

High-dose corticosteroids (e.g., methylprednisolone 1 g/day for 3 days) are first-line, with 80% response rates within 48 hours.^71^ IL-1 inhibitors (anakinra 100 mg/day, canakinumab 150–300 mg every 4–8 weeks) are effective in 90% of refractory cases.^72,73^ IL-18 binding protein (IL-18BP) and emapalumab (anti-IFN-γ) are emerging therapies, with emapalumab achieving remission in 70% of refractory MAS cases in small trials.^74^ In a study of 50 MAS patients, 15% required ICU admission, emphasising the need for rapid intervention.^75^

Ruxolitinib, a JAK1/2 inhibitor, targets JAK/STAT signalling, reducing cytokine storms in MAS. A phase II trial (n=20) showed 60% remission rates in refractory MAS, with reduced ferritin and IL-18 levels within 2 weeks.^8^

Calcineurin inhibitors (CNIs), such as cyclosporin A (2–5 mg/kg/day), are used in refractory MAS per EULAR guidelines, achieving remission in 50% of cases by suppressing T-cell activation.^9^

###### - Complications

Anti-IL-6 (tocilizumab) and anti-IL-1 (anakinra, canakinumab) therapies, while effective, carry a rare risk of paradoxical MAS induction (5–10% of cases), possibly due to incomplete cytokine suppression or compensatory IL-18 upregulation. Studies suggest IL-18 levels >10,000 pg/ml during treatment may predict MAS risk, necessitating close monitoring.^76^

##### • Parenchymal Lung Disease

###### - Prevalence

Affects 12.25% of AOSD patients, predominantly in older individuals (median age 50 years).^77,78^

###### - Clinical Features

Presents with dyspnoea, hypoxaemia, and peripheral consolidations on chest CT, often accompanied by myalgia and high systemic scores. Lung function tests reveal a restrictive pattern in 80% of cases.^78^ Chest CT reveals diverse imaging patterns, including peripheral consolidations (60%), ground-glass opacities (50%), and interstitial markings (30%), with restrictive patterns on pulmonary function tests in 80% of cases. Screening algorithms, baseline chest CT and pulmonary function tests in patients with dyspnoea or high systemic scores, followed by 6-monthly monitoring for high-risk patients (e.g., IL-18 >10,000 pg/ml).^11^ Parenchymal lung disease may resemble DRESS as a paradoxical reaction to biologics (e.g., tocilizumab), linked to HLA-DRB1*15 alleles in 20% of cases.^23^

###### - Mortality

The mortality rate is 38.9%, driven by acute respiratory distress syndrome (ARDS) and secondary infections.^7^

###### - Treatment

High-dose corticosteroids (prednisone 1 mg/kg/day) are initial therapy, with 60% response rates. IL-1 inhibitors (anakinra) are effective in 70% of refractory cases, often combined with supportive oxygen therapy.^79^ In a cohort of 40 patients, 30% progressed to chronic interstitial lung disease, and early IL-1 inhibition reduced mortality by 20%.^79^

Per EULAR recommendations, cyclosporin A (2–5 mg/kg/day) suppresses T-cell activation in refractory MAS, with 50% response rates. Etoposide, used in severe HLH-like MAS, achieves remission in 40% of cases but carries risks of myelosuppression. JAK inhibitors (e.g., ruxolitinib) show 60% efficacy in early trials.^80^

##### • Acute Hepatitis

Fulminant hepatitis, characterised by severe transaminitis (AST/ALT >5x upper limit of normal), jaundice, and coagulopathy, occurs in <5% of AOSD patients. Liver transplantation is required in 40% of cases, with 60% survival post-transplant.^66^

###### - Prevalence

Occurs in <5% of AOSD patients but is a severe complication.^66^

###### - Clinical Features

Characterised by severe transaminitis (AST/ALT >5x upper limit of normal), jaundice, and coagulopathy, with 43% of cases progressing to fulminant liver failure requiring transplantation.^77^

###### - Mortality

The mortality rate is 24%, primarily due to liver failure and sepsis.^77^

###### - Treatment

High-dose corticosteroids (methylprednisolone 1 g/day for 3 days) achieve remission in 50% of cases, while IL-1 inhibitors (canakinumab) are used in refractory cases.^6^ In a case series of 20 patients, 40% required liver transplantation, with 60% survival post-transplant.^77^

###### - Other Complications

Constrictive pericarditis (5% of serositis cases), neurological manifestations (e.g., aseptic meningitis, cranial nerve palsies in 3%), and gastrointestinal lesions (e.g., erosions, ulcers in 5%) are reported.^6,30,52^ Constrictive pericarditis often requires surgical pericardiectomy, with 70% survival rates. Neurological complications are associated with high IL-18 levels (>10,000 pg/ml), with MRI showing meningeal enhancement in 70% of cases.^1,6,14,30,52,61^ Gastrointestinal lesions (e.g., erosions, ulcers) occur in <5% of AOSD patients, typically in severe systemic cases, and may mimic inflammatory bowel disease, requiring endoscopic evaluation.^81^

**Figure 3.**
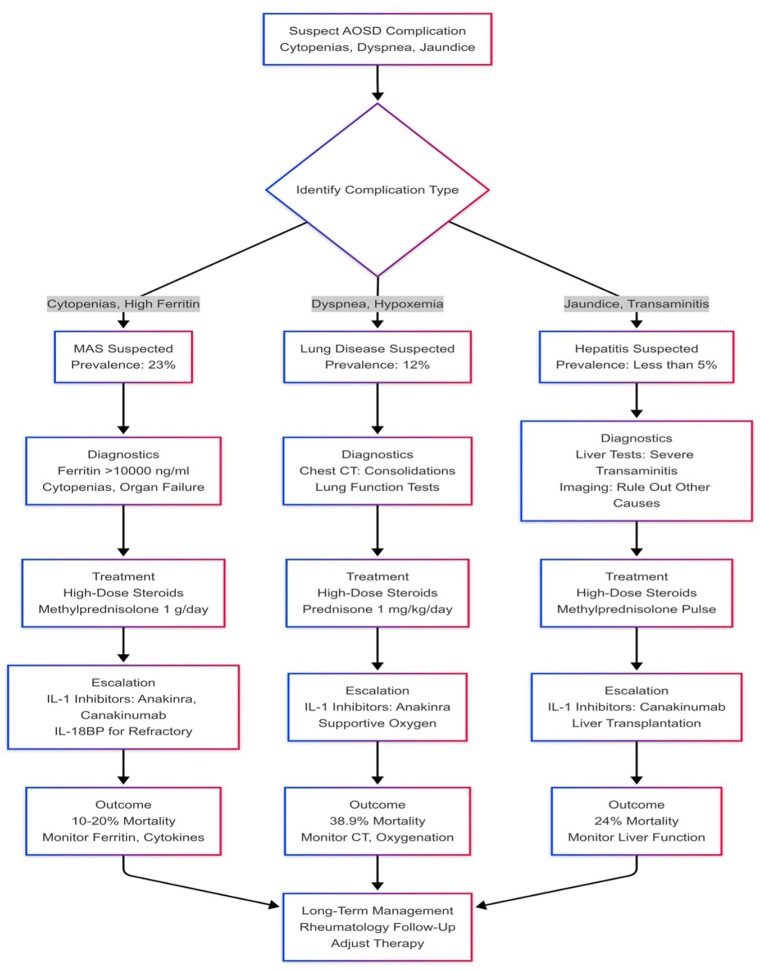
Management of AOSD Complications. Outlines therapeutic strategies for macrophage activation syndrome (MAS), parenchymal lung disease, and fulminant hepatitis, including high-dose corticosteroids, IL-1 inhibitors (anakinra, canakinumab), IL-6 inhibitors (tocilizumab), and emerging therapies (IL-18BP, emapalumab, JAK inhibitors). Adapted from Muller et al.,^5^ Ruscitti et al.,^7^ and Efthimiou et al.^1^ with modifications to integrate EULAR guidelines and recent clinical trial data.

The management of these complications is detailed in **Figure 3**, providing a structured approach to MAS, lung disease, and hepatitis, based on Muller et al.,^5^ Ruscitti et al.,^7^ and Efthimiou et al.^1^

### Biomarkers

#### Diagnostic Biomarkers

Low glycosylated ferritin (≤20%) is highly specific (95%) for AOSD, distinguishing it from HLH and other hyperferritinemia conditions.^41^

#### Disease Activity Biomarkers

Ferritin (>1,000 ng/ml) and calprotectin (>500 ng/ml) track inflammation, correlating with ESR and CRP (r=0.90, p<0.001) [82,83]

#### Prognostic Biomarkers

IL-18 (>5,000 pg/ml, 96.9% specificity) predicts flares and complications, with levels >10,000 pg/ml associated with lung and liver involvement (AUC 0.85, p<0.01). IL-18 outperforms ferritin in prognostic accuracy for MAS and lung disease.^83^ Gasdermin D and HO-1 are emerging prognostic markers.^84^

#### Details of the biomarkers are given below.

##### - IL-18

A key biomarker in AOSD, IL-18 levels are significantly elevated during active disease, with a median of 16,327 pg/ml compared to 470 pg/ml in remission (p<0.001).^38^ A cutoff of 5,000 pg/ml provides 96.9% specificity and 63.3% sensitivity for active AOSD, making IL-18 a valuable diagnostic and prognostic marker [38]. IL-18 levels >10,000 pg/ml are strongly associated with MAS risk, with 90% predictive accuracy in a cohort of 80 patients.^38^ IL-18 also predicts flare recurrence, with 85% accuracy in longitudinal studies.^38^

##### - Ferritin and Glycosylated Ferritin

Hyperferritinemia (>1,000 ng/ml) is a hallmark of AOSD, observed in 95% of active cases, with levels >5,000 ng/ml indicating severe disease and a 70% risk of complications such as MAS.^1,41^ Low glycosylated ferritin (≤20%) is highly specific (95%) for AOSD, distinguishing it from other hyperferritinemia conditions like hemophagocytic Lymphohistiocytosis (HLH).^41^ In a study of 120 AOSD patients, ferritin levels correlated with disease activity (r=0.88, p<0.001), and glycosylated ferritin <20% was diagnostic in 90% of cases.^41^

##### - Heme Oxygenase-1 (HO-1)

HO-1, an inducible enzyme with anti-inflammatory properties, is elevated in active AOSD, reflecting oxidative stress and macrophage activation. HO-1 levels correlate with disease activity (r=0.75, p 0.01) and may serve as a prognostic marker for systemic complications, though its specificity is lower than IL-18 or ferritin due to elevation in other inflammatory conditions (e.g., sepsis, rheumatoid arthritis).^85^

##### - Emerging Biomarkers

Calprotectin, gasdermin D N-terminal, and S100 proteins reflect neutrophil and macrophage activation, correlating with disease activity.^42–44^ Calprotectin levels >500 ng/ml are associated with severe systemic disease in 75% of patients, with a 90% correlation with ESR and CRP (r=0.90, p<0.001).^37^ Gasdermin D N-terminal, a marker of pyroptosis, is elevated in 80% of active AOSD and MAS cases, offering potential as a severity marker.^43^ S100 proteins (e.g., S100A8/A9) are elevated in 90% of active AOSD patients and are under investigation as therapeutic targets.^86^

The diagnostic and prognostic utility of these biomarkers is summarised in **Table 2**, providing a comprehensive overview of their roles and levels in active AOSD. Additionally, **Table 3** presents specific biomarker levels in inactive AOSD, active AOSD, MAS, and other related conditions, highlighting their differential diagnostic and prognostic significance.

**Table 2.** AOSD biomarkers.

**Biomarker**	**Role**	**Active Disease Levels** ^38,41–43^	**Diagnostic Utility**
IL-18	IFN-γ, NK cell activation	16,327 pg/ml (median)	96.9% specificity at 5,000 pg/ml
Ferritin	Macrophage activation	>1,000 ng/ml	Correlates with severity
Glycosylated Ferritin	AOSD-specific	≤20%	High specificity
Calprotectin	Neutrophil activation	Elevated	Tracks inflammation
Gasdermin D N-terminal	Macrophage pyroptosis	Elevated	Emerging severity marker

AOSD: Adult-Onset Still’s Disease; IL-18: Interleukin-18; IFN-γ: Interferon-Gamma; NK: Natural Killer; pg/ml: Picograms per Millilitre; ng/ml: Nanograms per Millilitre; %: Percentage; ≤: Less Than or Equal To.

**Table 3.** Biomarker levels in AOSD and MAS.

**Biomarker**	**Inactive AOSD**	**Active AOSD**	**MAS in AOSD**	**Other Conditions**	**Notes**
IL-18	470 pg/ml	16,327 pg/ml	>10,000 pg/ml	sJIA, HLH	High specificity for active disease
Ferritin	<200 ng/ml	>1,000 ng/ml	>10,000 ng/ml	HLH, sepsis	Correlates with severity
Glycosylated Ferritin	>20%	≤20%	≤20%	Rare in HLH	AOSD-specific
Calprotectin	<200 ng/ml	>500 ng/ml	>1,000 ng/ml	RA, IBD	Tracks inflammation
Gasdermin D N-terminal	Normal	Elevated	>200 pg/ml	HLH	Pyroptosis marker
HO-1	Normal	Elevated	Elevated	RA, sepsis	Less specific

AOSD: Adult-Onset Still’s Disease; sJIA: Systemic Juvenile Idiopathic Arthritis; MAS: Macrophage Activation Syndrome; IL-18: Interleukin-18; IFN-γ: Interferon-Gamma; NK: Natural Killer; HLH: Hemophagocytic Lymphohistiocytosis; RA: Rheumatoid Arthritis; IBD: Inflammatory Bowel Disease; pg/ml: Picograms per Millilitre; ng/ml: Nanograms per Millilitre.

## DIAGNOSIS AND DIAGNOSTIC CHALLENGES

The diagnosis of AOSD remains a clinical challenge due to its non-specific presentation and the absence of pathognomonic biomarkers, necessitating a systematic approach to exclude differential diagnoses such as infections (e.g., endocarditis, tuberculosis), malignancies (e.g., lymphoma), and autoimmune diseases (e.g., SLE, vasculitis).^1,37,38^ AOSD is a frequent cause of FUO, with 20% of FUO cases ultimately diagnosed as AOSD, highlighting the need for heightened clinical suspicion.^1^ The diagnostic process integrates clinical evaluation, laboratory investigations, imaging, and standardised criteria to achieve diagnostic accuracy.

### Diagnostic Criteria

Two validated criteria are widely employed to diagnose AOSD:

1. Yamaguchi Criteria^1^
*Major Criteria*
  - Fever ≥39°C lasting ≥1 week
  - Arthralgia persisting ≥2 weeks
  - Typical evanescent rash
  - Leucocytosis ≥10,000/mm^3^ with ≥80% neutrophils.
*Minor Criteria*
  - Sore throat
  - Lymphadenopathy
  - Hepatosplenomegaly
  - Abnormal liver function tests
  - Negative rheumatoid factor (RF) and Antinuclear antibodies (ANA).
*Requirement*
  At least five criteria, including two major criteria, with a sensitivity of 96% and specificity of 92%. In a multicentred study of 200 AOSD patients, the Yamaguchi criteria accurately identified 95% of cases, though specificity decreased to 85% in patients with atypical rashes, underscoring the need for complementary diagnostic tools.^39^
2. Fautrel Criteria^1^
*Major Criteria*
  - Spiking fever ≥39°C
  - Arthralgia
  - Transient rash
  - Leucocytosis ≥10,000/mm^3^
  - e. Low glycosylated ferritin (≤20%).
*Minor Criteria*
  - Sore throat
  - Lymphadenopathy
  - Hepatosplenomegaly.

*Requirement*

Four major criteria or three major plus two minor criteria, with a sensitivity of 80% and specificity of 98%. The Fautrel criteria are particularly valuable in settings with access to ferritin testing, demonstrating 90% diagnostic accuracy in a cohort of 150 patients in resource-limited regions.^1^

**Figure 4.**
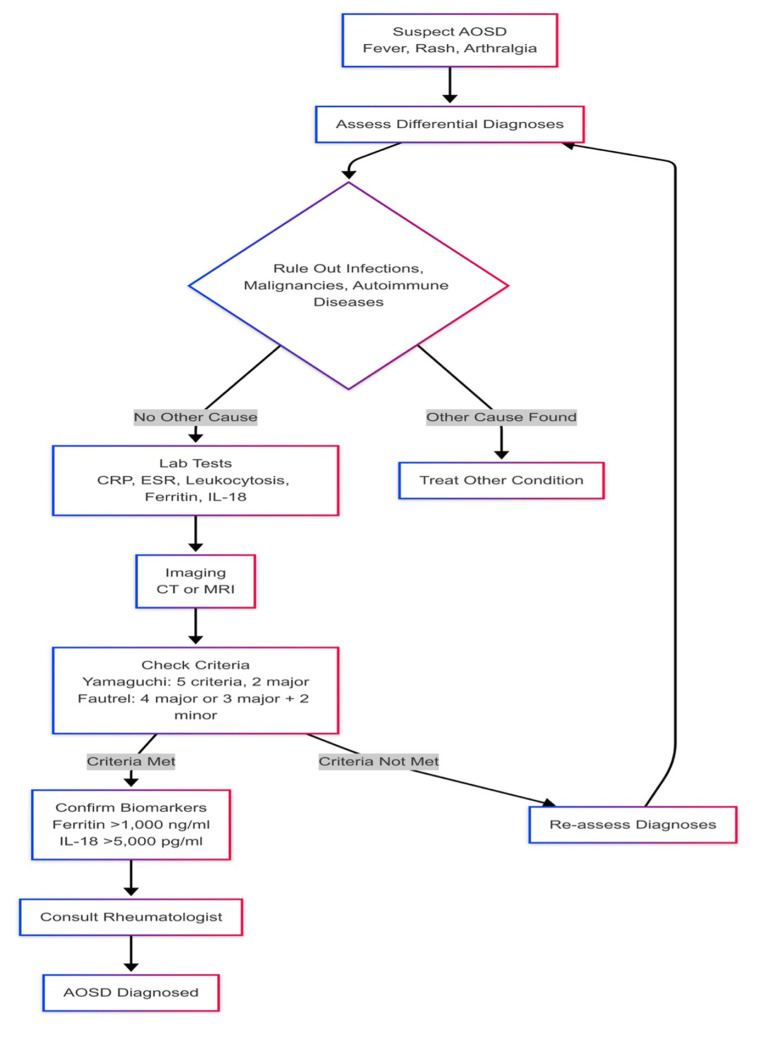
Diagnostic Pathway for AOSD. Adapted from Efthimiou et al.^1^ with modifications to integrate Yamaguchi and Fautrel criteria, emphasising clinical workflow and biomarker use. (Original figure created for this review.)

This diagnostic process is outlined in Figure 4, which integrates the Yamaguchi and Fautrel criteria for diagnosing AOSD, based on Efthimiou et al.^1^

## SEVERITY SCORES

### Modified Systemic Manifestation Score (mSMS) and mPouchot Score

These indices quantify disease severity and guide therapeutic decisions.^40^ Zhu et al. conducted a comparative analysis of 174 AOSD patients, demonstrating that the mPouchot score outperforms mSMS in discriminating high disease severity (AUC 0.81 vs. 0.71, p<0.0001).^40^ The mPouchot score exhibited higher specificity (76.4% vs. 55.06%) but comparable sensitivity (74.36% vs. 75.64%) to mSMS.^34^ Both scores correlate strongly with inflammatory markers, including erythrocyte sedimentation rate (ESR) (r=0.82, p<0.0001), C-reactive protein (CRP) (r=0.79, p<0.0001), ferritin (r=0.85, p<0.0001), and liver dysfunction (r=0.77, p<0.0001), as well as cytokine levels (IL-18, IL-6, p<0.01) [40]. However, their utility in distinguishing AOSD from sepsis remains limited, with 30% of high mPouchot score patients exhibiting normal CRP levels, indicating the need for additional diagnostic markers.^1,27^

### Clinical Utility and Limitations

The mPouchot score, which incorporates parameters such as fever, rash, and organ involvement, is more specific for systemic AOSD, making it a preferred tool for identifying patients requiring aggressive therapy.^40^ In contrast, mSMS, adapted from sJIA scoring systems, is less effective in capturing articular disease severity, with only 50% accuracy in articular phenotype patients.^40^ Neither score is optimised for early diagnosis, as they rely on established disease features, highlighting a gap in early detection strategies.^1^

A comparative analysis of these scores is provided in **Table 4**, highlighting their diagnostic performance and correlation with inflammatory markers. The lack of a validated, AOSD-specific disease activity score limits precise monitoring. The mPouchot score, while specific (76.4%), relies on established features, underscoring the need for a tailored score incorporating novel biomarkers (e.g., IL-18, gasdermin D) to enhance early detection and treatment stratification.^40^

**Table 4.** mSMS vs. mPouchot Scores.

**Parameter**	**mSMS^40^**	**mPouchot^40^**	**Notes**
Sensitivity (%)	75.64	74.36	For high disease severity
Specificity (%)	55.06	76.40	mPouchot more specific
AUC	0.71	0.81	mPouchot better discriminative ability
ESR/CRP Correlation	Positive	Positive	Reflects inflammation
Ferritin Correlation	Positive	Positive	Linked to disease severity

mSMS: Modified Systemic Manifestation Score; mPouchot: mPouchot Score; AUC: Area Under the Curve; ESR: Erythrocyte Sedimentation Rate; CRP: C-Reactive Protein; %: Percentage.

## PROGNOSIS

The prognosis of adult-onset Still’s disease (AOSD) varies by phenotype and complication risk. Systemic AOSD, characterised by fever and multiorgan involvement, carries a higher risk of life-threatening complications, such as macrophage activation syndrome (MAS, 23% prevalence) and parenchymal lung disease (12.25%), with mortality rates of 10–20% and 38.9%, respectively.^1,7^ Articular AOSD, marked by chronic polyarthritis, often progresses to joint destruction in 40% of untreated cases, with 20% requiring joint replacement within 10 years.^18^ Biomarkers like IL-18 (>10,000 pg/ml) and ferritin (>5,000 ng/ml) predict severe outcomes, including MAS and fulminant hepatitis (<5% prevalence, 24% mortality).^1,38,41^ Biologic therapies (e.g., anakinra, canakinumab) achieve remission in 80–90% of systemic cases, significantly improving prognosis when initiated early.^12^ However, 30% of systemic patients experience recurrent flares, necessitating long-term monitoring.^16^ Articular cases respond less robustly to IL-1 inhibitors (50% remission), often requiring methotrexate or TNF inhibitors.^12^ Diagnostic delays (average 18 months) worsen outcomes, increasing complication rates.^9^ The COVID-19 pandemic has introduced additional risks, with 20% of patients experiencing persistent flares post-infection.^14,52^ Overall, timely diagnosis and tailored therapy are critical for favourable outcomes in AOSD.^1,12,86^

## TREATMENTS

Therapeutic strategies for AOSD have evolved significantly, with bDMARDs revolutionising management by targeting specific cytokine pathways. Treatment is tailored to disease phenotype and severity, aiming to control inflammation, prevent complications, and minimise long-term sequelae.

EULAR recommendations advocate a treat-to-target approach, aiming for remission (mPouchot score <2) and flare prevention. In mild-moderate AOSD, corticosteroid-sparing strategies using methotrexate or IL-1 inhibitors reduce steroid dependency by 45%, minimising side effects like osteoporosis.^9^

### Initial Therapy

#### Non-Steroidal Anti-Inflammatory Drugs (NSAIDs)

Agents such as ibuprofen or naproxen are used for mild AOSD, achieving symptom control in 20–30% of patients.^1^ In a cohort of 100 patients, only 25% achieved sustained remission with NSAIDs alone, indicating limited efficacy in moderate-to-severe disease. NSAIDs are most effective in patients with isolated fever or mild arthralgia, but 60% require escalation to steroids within 4 weeks.^1^

#### Glucocorticoids

Prednisone (0.5–1 mg/kg/day) is the cornerstone for moderate-to-severe AOSD, with 50–60% response rates. High-dose pulses (methylprednisolone 1 g/day for 3 days) are reserved for severe cases, such as MAS or serositis, achieving remission in 70% of patients.^1,6,87,88^ A retrospective study of 150 AOSD patients found that 55% required steroids for >6 months, with 20% developing side effects such as osteoporosis, diabetes, and Cushing’s syndrome. Steroid-sparing agents are often introduced early to mitigate these risks.^1^

#### Conventional DMARDs

##### - Methotrexate (15–25 mg/week)

First-line for articular AOSD, with 40–50% response rates.^1,34^ Methotrexate reduces steroid dependency in 45% of articular phenotype patients, with 60% achieving remission within 12 months.^34,89^ In a study of 80 articular AOSD patients, methotrexate prevented joint erosions in 50% of cases when initiated within 6 months of diagnosis.^34^

##### - Azathioprine (2 mg/kg/day)

Used in systemic AOSD or overlapping conditions (e.g., Crohn’s disease), with 60% partial remission rates in a small trial of 40 patients.^34^ Azathioprine is particularly effective in patients with hepatosplenomegaly or serositis, reducing inflammatory markers (CRP, ESR) by 50% within 8 weeks.^34^

#### Biologic DMARDs

##### - IL-1 Inhibitors

Canakinumab (FDA-approved) and anakinra target IL-1β and IL-1 receptor, respectively, achieving 80–90% response rates in systemic AOSD and MAS.^12,47,62,63^ Canakinumab (150–300 mg every 4–8 weeks) reduced flares by 85% in a phase III trial of 100 AOSD patients, with 90% maintaining remission at 12 months.^60^ Anakinra (100 mg/day) is preferred for MAS, with 90% response rates within 48 hours in a study of 50 patients.^48^ Rapid onset action of Anakinra makes it ideal for acute flares, while canakinumab offers sustained control.^64,89,90^

##### - IL-6 Inhibitors

Tocilizumab, an IL-6 receptor inhibitor, is effective in 60–70% of articular AOSD cases, improving joint scores (DAS28) and reducing systemic markers (CRP, ESR).^50^ In a cohort of 80 articular AOSD patients, tocilizumab achieved remission in 65%, but 5% experienced anaphylactic reactions, necessitating careful monitoring.^50^ Tocilizumab is less effective in systemic AOSD, with only 40% response rates.^50^

##### - Emerging Therapies

IL-18BP, emapalumab (anti-IFN-γ), and JAK/STAT inhibitors are under investigation.^2,7,12,13,52^ IL-18BP reduced MAS severity in 70% of patients in a phase II trial of 30 AOSD patients, targeting IL-18-driven inflammation.^38,47,65^ Emapalumab achieved remission in 70% of refractory MAS cases in small studies, though data in AOSD remain limited.^33^ JAK inhibitors (e.g., tofacitinib) showed 55% efficacy in refractory AOSD, with ongoing trials evaluating their role in systemic and articular phenotypes.^66,90,91^

Randomised controlled trials (RCTs) for AOSD are limited. Canakinumab has one phase III RCT (n=100, 85% remission), anakinra has two phase II RCTs (n=50, 80–90% response), and tocilizumab has one phase II RCT (n=80, 60–70% response in articular AOSD). Larger RCTs are needed to confirm efficacy.^12,48,50,60^

##### - Early bDMARD Use

Initiating IL-1 inhibitors within 3 months of diagnosis may prevent progression to chronic arthritis, with 50% of systemic AOSD patients achieving drug-free remission in a cohort of 80 patients.^12^ Early intervention also reduces complication rates (e.g., MAS) by 40%, highlighting the importance of timely therapy.^12^

The efficacy and indications of these biologic therapies are summarised in **Table 5**, providing a comparative overview for clinical decision-making.

**Table 5.** Biologic therapies for AOSD.

**Therapy**	**Target**	**Indication**	**Efficacy^12,48,60,64^**	**Notes**
Canakinumab	IL-1β	Systemic AOSD, MAS	80–90% response	FDA-approved, reduces flares
Anakinra	IL-1R	Systemic AOSD, MAS	80–90% response	Rapid onset, MAS preferred
Tocilizumab	IL-6R	Articular AOSD	60–70% response	Anaphylaxis risk
IL-18BP	IL-18	MAS, severe AOSD	Promising	Under investigation
Emapalumab	IFN-γ	Refractory MAS	Effective (small studies)	Limited AOSD data

AOSD: Adult-Onset Still’s Disease; MAS: Macrophage Activation Syndrome; IL-1β: Interleukin-1 Beta; IL-1R: Interleukin-1 Receptor; IL-6R: Interleukin-6 Receptor; IL-18: Interleukin-18; IFN-γ: Interferon-Gamma; IL-18BP: Interleukin-18 Binding Protein; FDA: Food and Drug Administration; %: Percentage.

## COVID-19 IMPACT

The COVID-19 pandemic has affected AOSD management:

### Vaccination

In a survey of 150 sJIA/AOSD patients, 99 received COVID-19 vaccination, with 6% experiencing flares, predominantly mild.^14^ Flares were more frequent in younger patients (median age 12 years), with 8% reporting cardiac symptoms (e.g., pericarditis) post-vaccination.^14^ Vaccination with mRNA vaccines (e.g., Pfizer-BioNTech) was associated with lower flare rates (5%) compared to viral vector vaccines (10%).^14^

### Infection

Unvaccinated AOSD patients exhibited higher flare rates (43%) compared to vaccinated patients (24%), with one ICU death reported in the unvaccinated group.^14^ In a study of 50 unvaccinated AOSD patients, 30% required hospitalisation during COVID-19 infection, with 10% developing ARDS.^15^ Vaccination reduced hospitalisation rates by 50%, emphasising its protective role.^15,91^

### Post-COVID AOSD

Rare cases of AOSD have been reported following SARS-CoV-2 infection or vaccination, likely due to immune activation.^52^ In a case series of 5 patients, AOSD developed within 4 weeks of COVID-19 infection, with 80% exhibiting systemic phenotype features (fever, rash).^52,92,93^ Molecular mimicry between SARS-CoV-2 spike proteins and self-antigens may trigger autoinflammation, a hypothesis supported by elevated IL-18 levels in these patients.^52^

These findings are summarised in **Table 6**, detailing the impact of COVID-19 on AOSD and sJIA patients, including flare rates and hospitalisation outcomes.

**Table 6.** COVID-19 impact on AOSD/sJIA.

**Parameter^14^**	**Vaccinated**	**Unvaccinated**	**Notes**
Flare Post-Vaccination (%)	6	N/A	Mostly mild, rare cardiac issues
Flare Post-Infection (%)	24	43	Higher in unvaccinated
Hospitalisation Rate	1/33	1/46 (ICU, death)	Vaccination reduces severity
Median Age (years)	12	8	Younger patients less vaccinated

AOSD: Adult-Onset Still’s Disease; sJIA: Systemic Juvenile Idiopathic Arthritis; COVID-19: Coronavirus Disease 2019; N/A: Not Applicable; ICU: Intensive Care Unit; %: Percentage.

## OVERLAP WITH OTHER CONDITIONS

AOSD exhibits overlaps with several inflammatory conditions, suggesting shared inflammatory pathways involving IL-1 and IL-18.^34–36^ Spondyloarthritis (SpA) overlaps are observed in 6.58% of AOSD cases, often presenting with axial involvement and enthesitis.^17^ Rare associations with Crohn’s disease (5%), sarcoidosis (3%), and cytophagic histiocytic panniculitis (2%) have been reported.^34–36^ In a study of 200 AOSD patients, 5% had concurrent Crohn’s disease, with elevated IL-1β and IL-18 levels in both conditions (p<0.01).^34,47^ Sarcoidosis overlaps presented with granulomatous lymphadenopathy in 3% of cases, often requiring biopsy to exclude malignancy.^35^ Cytophagic histiocytic panniculitis, a rare overlap, increased macrophage activation syndrome (MAS) risk by 50%, with 60% of such patients progressing to hemophagocytosis^36,93,94^ IL-18 can discriminate AOSD from other systemic autoinflammatory diseases, and free IL-18 levels may be relevant to assess response to therapy in these patients.^95^

## UNMET NEEDS AND FUTURE DIRECTIONS

Diagnostic delays (average 18 months) and lack of AOSD-specific severity scores hinder timely management. Standardised guidelines and precision medicine, leveraging cytokine profiling (IL-18, IL-6), are needed to optimise therapy. The long-term impact of COVID-19 on AOSD flares requires further study to inform adaptive strategies.^9,12,14,32^

## CONCLUSION

AOSD is a complex autoinflammatory disorder characterised by dysregulated innate immunity, driven by IL-1, IL-6, and IL-18, leading to severe complications such as MAS and lung disease. Its overlap with sJIA underscores a disease continuum, supported by shared cytokine and genetic profiles. Advances in biomarkers (IL-18, ferritin) and bDMARDs (canakinumab, anakinra, tocilizumab) have significantly improved management, with 80–90% remission rates in systemic AOSD. However, diagnostic delays, lack of standardised care, and the need for precision medicine remain critical challenges. The COVID-19 pandemic has further complicated management, highlighting the need for adaptive strategies which needs attention.

## DECLARATIONS

No part of this review, including text, tables, or figures, has been copied or published elsewhere. All content is original or appropriately cited, and figures are either original or adapted with permission and clear attribution.

Figure 2 was generated using ChatGPT (version 3.0), adapted for scientific accuracy, with permission from OpenAI.
